# Comparative features and outcomes of cardiogenic shock in patients with and without prior resuscitated shockable cardiac arrest: Insight from the FRENSHOCK multicenter prospective registry

**DOI:** 10.1016/j.resplu.2025.101024

**Published:** 2025-07-09

**Authors:** Hamid Merdji, Vincent Bataille, Anais Curtiaud, Laurent Bonello, François Roubille, Bruno Levy, Pascal Lim, Jean-Claude Dib, Julien Maizel, Nicolas Brechot, Marion Beuzelin, Emmanuelle Fillippi, Miloud Cherbi, Julien Demiselle, Grégoire Rangé, Jérémie Joffre, Marwan Yassine, Caroline Biendel, Fanny Bounes, Guillaume Leurent, Edouard Gerbaud, Eric Bonnefoy, Etienne Puymirat, Clément Delmas, FRENSHOCK Investigators, Nadia Aissaoui, François Bagate, Marion Beuzelin, Caroline Biendel, Florence Boissier, Laurent Bonello, Éric Bonnefoy-Cudraz, Marie Boughenou, Stéphane Boule, Jérémie Bourenne, Nicolas Brechot, Cédric Bruel, Alain Cariou, Philippe Castellant, Sébastien Champion, Karim Chaoui, Marion Chatot, Nicolas Combaret, Nicolas Debry, Xavier Delabranche, Jean-Claude Dib, Raphael Favory, Emmanuelle Filippi, Romain Gallet, Frédérique Ganster, Philippe Gaudard, Edouard Gerbaud, Brahim Harbaoui, Patrick Henry, Benoit Herce, Fabrice Ivanes, Jérémie Joffre, Philippe Karoubi, Hadi Khachab, Khalifé Khalife, Kada Klouche, Vincent Labbe, Marc Laine, Nicolas Lamblin, Benoit Lattuca, Yann Lefetz, Gilles Lemesle, Philippe Letocart, Leurent, Bruno Levy, Guillaume Louis, Julien Maizel, Jacques Mansourati, Stéphane Manzo-Silberman, Séverine Marchand, Benjamin Marchandot, Stéphanie Marliere, Joy Mootien, Frédéric Mouquet, Louis Niquet, Alexis Paternot, Vincent Probst, Etienne Puymirat, Charlotte Quentin, Grégoire Range, Nassim Redjimi, Jean Christophe Richard, François Roubille, Christophe Saint Etienne, Francis Schneider, Guillaume Schurtz, Marie-France Seronde, Julien Ternacle, Gérald Vanzetto, Elie Zogheib

**Affiliations:** a Université de Strasbourg (UNISTRA), Faculté de Médecine, Strasbourg University Hospital, Nouvel Hôpital Civil, Medical Intensive Care Unit, Strasbourg, France; b Department of Cardiology, Toulouse Rangueil University Hospital, UMR 1295 INSERM, Toulouse, France; c Association pour la Médecine de Prévention, Toulouse, France; d Aix-Marseille Université, F-13385 Marseille, France; e Intensive Care Unit, Department of Cardiology, Assistance Publique-Hôpitaux de Marseille, Hôpital Nord, F-13385 Marseille, France; f Mediterranean Association for Research and Studies in Cardiology (MARS Cardio), Marseille, France; g PhyMedExp, Université de Montpellier, INSERM, CNRS, Cardiology Department, INI-CRT, CHU de Montpellier, France; h CHRU Nancy, Réanimation Médicale Brabois, Vandoeuvre-les Nancy, France; i Univ Paris Est Créteil, INSERM, IMRB, F-94010 Créteil, France; j AP-HP, Hôpital Universitaire Henri-Mondor, Service de Cardiologie, F-94010 Créteil, France; k Clinique Ambroise Paré, Neuilly-sur-Seine, France; l Service de Médecine Intensive Réanimation, Centre Hospitalier Universitaire Amiens-Picardie, Amiens, Hauts-de-France, France; m Service de Médecine Intensive-Réanimation, Hôpital Européen George-Pompidou, Université Paris Descartes, AP-HP Paris, France; n Intensive Care Unit, Centre Hospitalier de Dieppe, Dieppe, France; o Service de Cardiologie, CH de Vannes 56000 Vannes, France; p Intensive Cardiac Care Unit, Rangueil University Hospital, 31059 Toulouse, France; q Institute of Metabolic and Cardiovascular Diseases (I2MC), Inserm UMR-1048, 31432 Toulouse, France; r Cardiology Department, Centre Hospitalier Louis-Pasteur, 28630 Chartres, France; s Service de Réanimation Médicale, Hôpital de Saint Antoine, Assistance Publique-Hôpitaux de Paris (AP-HP), Paris, France; t Cardiology Department, Centre Hospitalier Metz-Thionville, 57530 Ars-Laquenexy, France; u Critical Care Unit, University Teaching Hospital of Rangueil, F-31400 Toulouse Cedex 9, France; v Department of Cardiology, CHU Rennes, Inserm, LTSI—UMR 1099, Univ Rennes 1, F-35000 Rennes, France; w Intensive Cardiac Care Unit and Interventional Cardiology, Hôpital Cardiologique du Haut Lévêque, 5 Avenue de Magellan, 33600 Pessac, France; x Bordeaux Cardio-Thoracic Research Centre, U1045, Bordeaux University, Hôpital Xavier Arnozan, Avenue du Haut Lévêque, 33600 Pessac, France; y Intensive Cardiac Care Unit, Lyon Bron University Hospital, Lyon, France; z Assistance Publique-Hôpitaux de Paris (AP-HP), Hôpital Européen Georges Pompidou, Department of Cardiology, Université de Paris 75006 Paris, France; aa Recherche et Enseignement en Insuffisance Cardiaque Avancée Assistance et Transplantation (REICATRA), Institut Saint Jacques, CHU Toulouse, France

**Keywords:** Heart failure, Cardiogenic shock, Cardiac arrest, Post-cardiac arrest syndrome

## Abstract

**Aim:**

Differences between cardiogenic shock (CS) with and without prior resuscitated cardiac arrest (CA) remain largely unexplored. We hypothesized that patients who experience shockable CA followed by CS are likely to have worse outcomes compared to CS without prior CA.

**Methods:**

FRENSHOCK is a prospective multicenter observational registry conducted in French critical care units in 2016, which included CS from various etiologies. Patients admitted after resuscitation of a CA were included if they fulfilled previously defined CS criteria. Non-shockable rhythms at the time of medical intervention were considered exclusion criteria and were not recorded in the registry.

**Results:**

Among the 771 enrolled patients (mean age 65.7 ± 14.9 years; 71.5 % male), 79 (10.2 %) had a resuscitated shockable cardiac arrest just before inclusion. Shockable CA patients had more respiratory support (78.5 % vs. 33.2 %, *p* < 0.001), more mechanical circulatory support (35.4 % vs. 16.5 %, *p* < 0.001), more coronary angiography performed (76 % vs. 48.8 %, *p* < 0.001), finding more mono-troncular lesions (39 % vs. 16.9 %, *p* < 0.001). Thirty-day and one-year survival were similar between groups. Among 30-day survivors, CS with an initial shockable CA exhibited significantly improved long-term survival compared to CS without prior resuscitated CA.

**Conclusion:**

In a cohort of patients with cardiogenic shock from various etiologies, approximately 10% had experienced prior resuscitation following a cardiac arrest with shockable rhythms. Our findings suggest that selected cardiac arrest with a shockable rhythm leading to cardiogenic shock does not inherently confer a worse prognosis compared to other causes of cardiogenic shock.

## Graphical abstract

**Figure:**
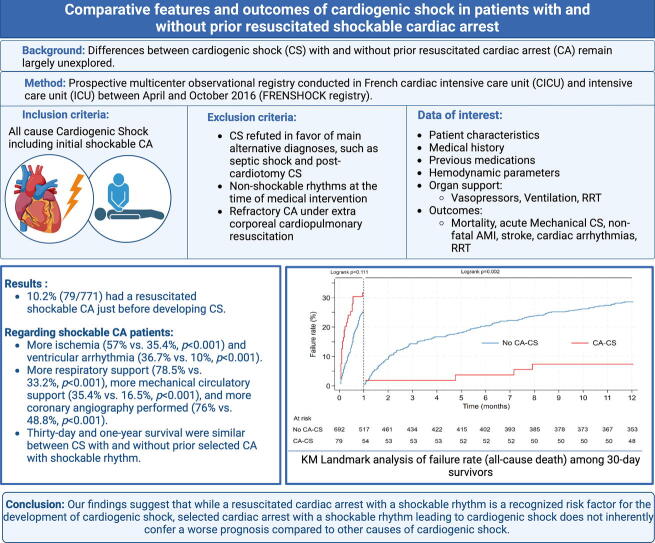

## Introduction

Cardiogenic shock (CS) is one of the most lethal complications of acute cardiovascular disease, with in-hospital mortality rates exceeding 30 % to 40 %.^1^ Similarly, cardiac arrest (CA) has a very high fatality rate, with nearly 90 % of out-of-hospital cardiac arrest (OHCA) patients in the U.S. not surviving, despite receiving assistance.^2^ Among those admitted to the hospital after OHCA, mortality remains high, at approximately 40 % to 50 %.^3^ Thus, both CS and CA are a significant burden on healthcare systems despite advances in supportive care.^4^

CS can result from a variety of etiologies, with resuscitated CA frequently occurring as one of its precipitating events, regardless of whether the CA is directly triggered by an initial myocardial issue. Consequently, many studies and clinical trials on CS encompass patients who develop CS following resuscitated CA, such as the IMPRESS in Severe Shock trial (which included 92 % patients with prior CA),^5^ the CULPRIT-SHOCK trial (>50 % of patients with prior CA),^6^ the HYPO-ECMO trial (almost 50 % patients with prior CA),^7^ the ECLS-SHOCK trial (>77 % patients with prior CA),^8^ and the DanGer Shock trial (almost 20 % of patients with prior CA).^9^ However, while CS in patients with and without preceding CA may share underlying myocardial substrates, their pathophysiological mechanisms and clinical outcomes often differ significantly. Indeed, patients with CS following resuscitated CA (CA-CS) frequently develop a complex post-resuscitation shock syndrome characterized by overlapping cardiogenic and distributive shock components, primarily attributed to ischemia–reperfusion injury and systemic inflammatory response syndrome (SIRS).^10^ This post-resuscitation shock introduces a complex clinical picture that differs markedly from that of non-CA-CS patients, who primarily experience shock due to pump failure. Additionally, CA-CS patients are more prone to develop anoxic brain injury, a severe neurological complication, which further distinguishes them from those with non-CA-CS.^11^

Exploring these differences is crucial for tailoring therapeutic strategies to the specific needs of each patient subgroup for redefining critical illness^12^. As such, distinguishing between CA-CS and non-CA-CS is essential for the design of future studies and for optimizing clinical management in this high-risk population.^13^, ^14^, ^15^ We hypothesized that patients who experience shockable CA followed by CS are likely to have worse outcomes than CS without prior CA. Therefore, our study aimed to assess the features and outcomes of CS in patients based on whether CA precedes the onset of CS. Using data from a dedicated CS registry, we aimed to compare the clinical characteristics, pathophysiological mechanisms, and outcomes between patients with CS following CA with a shockable rhythm (shockable CA-CS group) and those who develop CS without prior CA (non-CA-CS group). By clarifying these differences, we aim to provide essential insights that will enhance the understanding of these characteristics and contribute valuable data to more accurately assess the relevance of including CA-CS patients in future CS studies. The initial primary objective of the FRENSHOCK registry was to evaluate the characteristics, management, and outcomes of CS patients, with a new modified definition of CS as seen in routine clinical practice, on a nationwide scale.

## Methods

### Patient population

FRENSHOCK is a prospective multicenter observational registry conducted in metropolitan France during six months between April and October 2016 in intensive care unit (ICU) and cardiac intensive care unit (CICU) (NCT02703038). The methods used for this registry have been previously described.^16^ All adult patients (≥18 years old) with CS were prospectively included in this registry if they met at least one criterion of each of the following three components: (i) hemodynamic criteria, defined as low systolic blood pressure (SBP) < 90 mmHg and/or the need for maintenance with vasopressors/inotropes and/or a low cardiac index < 2.2 L/min/m^2^; (ii) left and/or right heart overload, defined by clinical signs, radiology, blood tests, echocardiography, or invasive hemodynamics’ signs; and (iii) signs of organ malperfusion (clinical and/or biological criteria). Patients could be included regardless of CS etiology, and whether CS was primary or secondary. Exclusion criteria were refusal or the inability to consent, and when a diagnosis of CS was refuted in favor of main alternative diagnoses, such as septic shock and post-cardiotomy CS.

Patients admitted after resuscitation of a CA were included only if they had a shockable rhythm and fulfilled previously defined CS criteria. Non-shockable rhythms at the time of medical intervention and refractory CA under extracorporeal cardiopulmonary resuscitation were considered as exclusion criteria and, therefore, were not included in the registry.

All institutions were invited to participate in the study, including university teaching hospitals, general and regional hospitals, and public and private hospitals that manage CS patients (CICUs, surgical ICUs, medical ICUs, and general ICUs). This inclusive approach was designed to capture a strong representation of adult cardiogenic shock admissions across France during the study period.

### Ethics

The study was conducted in accordance with the guidelines for good clinical practice and French law. Written consent was obtained for all the patients. The data recorded and their handling and storage were reviewed and approved by the CCTIRS (French Health Research Data Processing Advisory Committee) (no. 15.897) and the CNIL (French Data Protection Agency) (no. DR-2016-109).

### Data collection

The protocol for data collection has been published previously.^16^, ^17^ Briefly, the data collected included the patient’s medical history, past treatments, and management of CS during hospitalization, including fluid administration and the use of antibiotics, inotropes/vasopressors, mechanical ventilation, renal replacement therapy, and temporary mechanical circulatory support (tMCS). Additionally, various clinical, biological, and echocardiographic variables were recorded at admission and after 24 h. This protocol has been written according to the “Strengthening The Reporting of OBservational Studies in Epidemiology” (STROBE) guidelines.

### Follow-up and censoring

All patients included in the registry were followed for survival status up to one year after inclusion. In cases where vital status was not available at one year, patients were censored at the date of last known follow-up. Loss to follow-up was handled using appropriate time-to-event methods (Kaplan-Meier survival analyses and Cox proportional hazards models).

### Statistical analysis

Continuous variables were reported as means (SD) or medians and interquartile ranges when appropriate. Discrete variables were described in numbers and percentages. Groups (shockable CA-CS vs non-CA-CS) were compared using student’s *t*-tests (or non-parametric Mann & Whitney tests when skewed) for continuous variables; and *χ*^2^ or Fisher’s exact tests for qualitative variables. Survival in the two groups was computed using the Kaplan-Meier method, and the crude *(unadjusted)* difference in survival between the two groups was tested using the Logrank test. However, since the survival curves of patients with and without prior CA crossed between one and two months of follow-up, a landmark analysis was performed with a landmark set at 30 days. This approach allows for the comparison of long-term mortality (up to one year) among patients who survived beyond the initial 30 days. Additionally, a stratified analysis based on the variable shockable CA-CS vs. non-CA-CS was conducted to identify factors potentially associated with 30-day mortality in each group. This analysis was performed using a Cox proportional hazards regression model with a stepwise backward selection procedure. The initial model included all factors previously identified as associated with 30-day mortality in univariable analysis (with *p* < 0.15), adjusted for age. Only variables with *p* < 0.05 were retained in the final model. Although not strictly “crude” estimates, age-adjusted hazard ratios were derived from univariable Cox regression models adjusted solely for age, given its clinical relevance as a potential confounder. Only variables with *p* < 0.05 were retained in the final model. The assumption of the Cox model was assessed using Schoenfeld residuals and log(−log) survival plots, and first-order interaction terms were tested. For the CA-CS group, the initial model included the following variables: age, ongoing smoking, furosemide treatment, “other” trigger, sinus rhythm, estimated glomerular filtration rate (eGFR), left ventricular ejection fraction (LVEF), severe aortic stenosis, bilirubin, and BNP/Nt-proBNP. And for the non-CA-CS group, the initial variables were: age, diabetes, chronic kidney disease, ongoing treatment with either vitamin K antagonists or direct oral anticoagulants, infectious trigger, “other” trigger, admission unit, heart rate, systolic blood pressure, mottling, lactate levels, aspartate aminotransferase (AST), BNP/Nt-proBNP, C-reactive protein (CRP), and LVEF.

Statistical analyses were performed using Stata (StataCorp, 2023. Stata 18. Statistical software. StataCorp LLC. Two-sided *P* values <0.05 were considered significant for all tests.

## Results

### Study population

A total of 771 patients with CS were included across 49 centers, of whom 79 (10.2 %) had a CA just before admission. Clinical characteristics of patients with and without prior resuscitated CA before the onset of CS are summarized in Table 1**.** The mean age of the shockable CA-CS group was 60.8 ± 16.8 years, with a predominance of men (84.8 %). These characteristics differed significantly from the non-CA-CS group, where patients were older (66.3 ± 14.6) and less frequently represented by men (69.9 %). Shockable CA-CS patients were more frequently current smokers (48.6 % vs. 25.6 %, *p* < 0.001) and had a lower prevalence of pre-existing cardiac disease (38 % vs. 58.2 %, *p* = 0.004), likely contributing to fewer implanted defibrillators (2.5 % vs. 18.1 %, *p* = 0.003) and less use of cardiotropic medications, including aspirin, furosemide, aldosterone antagonists and vitamin K antagonists. This difference remained statistically significant after adjusting for age.

**Table 1:** Baseline characteristics of cardiogenic shock patients included.

		Overall (*n* = 771)		Non-CA-CS (*n* = 692)		CA-CS (*n* = 79)		Age-adj. *p*
Male gender		551	71.5	484	69.9	67	84.8	0.010
Age (years), mean ± SD		65.7	±14.9	66.3	±14.6	60.8	±16.8	−
BMI (kg/m^2^), mean ± SD		25.8	±5.5	25.8	±5.7	26.6	±4.4	0.283
	*n*	*744*		*668*		*76*		
Risk factors, *n* (%)								
	Current smoker	206	27.9	171	25.6	35	48.6	0.001
	Diabetes mellitus	217	28.2	201	29.1	16	20.3	0.264
	Arterial hypertension	364	47.2	327	47.3	37	46.8	0.156
	Dyslipidaemia	277	35.9	253	36.6	24	30.4	0.697
Medical history, *n* (%)								
	History of cardiac disease	433	56.2	403	58.2	30	38.0	0.004
	Ischaemic	230	29.8	211	30.5	19	24.1	0.664
	Hypertrophic	11	1.4	11	1.6	0	0.0	−
	Idiopathic	78	10.1	73	10.6	5	6.3	0.210
	Toxic	34	4.4	34	4.9	0	0.0	−
	Multisite pacing	63	8.2	61	8.8	2	2.5	0.112
	Defibrillator	127	16.5	125	18.1	2	2.5	0.003
	CABG	62	8.1	57	8.3	5	6.3	0.841
	PCI	166	21.6	150	21.7	16	20.3	0.695
	Peripheral artery disease	91	11.8	85	12.3	6	7.6	0.364
	Ischemic stroke	61	7.9	54	7.8	7	8.9	0.553
	Chronic renal failure	164	21.3	155	22.4	9	11.4	0.098
	Dialysis	11	1.4	10	1.5	1	1.3	0.842
	COPD	50	6.5	48	7.0	2	2.5	0.219
	Cancer	51	6.6	48	7.0	3	3.8	0.364
Previous medications before admission, *n* (%)								
	Aspirin	288	37.4	250	36.1	38	48.7	0.006
	P2Y12 inhibitor	126	16.4	113	16.3	13	16.7	0.733
	Statins	286	37.1	260	37.6	26	33.3	0.966
	Betablockers	316	41.0	291	42.1	25	32.1	0.184
	Vitamin K antagonist	165	21.4	159	23.0	6	7.7	0.008
	Direct oral anticoagulant	56	7.3	55	8.0	1	1.3	0.067
	ACE inhibitors or ARB	292	37.9	268	38.7	24	30.8	0.366
	Sacubitril/valsartan	18	2.5	18	2.8	0	0.0	−
	Furosemide	376	48.8	358	51.7	18	23.1	<0.001
	Aldosterone antagonist	108	14.0	107	15.5	1	1.3	0.007
	Amiodarone	132	17.6	124	18.4	8	10.4	0.100
	Proton pump inhibitor	276	36.4	259	38.0	17	22.1	0.019
Triggers								
	Ischaemic	290	37.6	245	35.4	45	57.0	<0.001
	Mechanical	23	3.0	22	3.2	1	1.3	0.424
	Ventricular arrhythmia	98	12.7	69	10.0	29	36.7	<0.001
	Atrial arrhythmia	108	14.0	100	14.5	8	10.1	0.285
	Conductive disorders	18	2.3	14	2.0	4	5.1	0.074
	Infectious	96	12.5	94	13.6	2	2.5	0.013
	Non compliance	30	3.9	29	4.2	1	1.3	0.229
	Iatrogenic	60	7.8	59	8.5	1	1.3	0.043
	Other	102	13.2	95	13.7	7	8.9	0.090
	None/undefined	111	14.4	106	15.3	5	6.3	0.054

ACE: Angiotensin-Converting Enzyme. ARB: Angiotensin-Receptor Blocker. BMI: Body Mass Index. CABG: Coronary Artery Bypass Graft. COPD: Chronic Obstructive Pulmonary Disease. PCI: Percutaneous Coronary Intervention. SD: Standard Deviation.

Regarding the triggers of CS (not mutually exclusive), ischemia (57 % vs. 35.4 %, *p* < 0.001) and ventricular arrhythmia (36.7 % vs. 10 %, *p* < 0.001) were significantly more common as etiologies in shockable CA-CS patients. Conversely, infection or iatrogenic factors were less frequent triggers of CS among CA-CS patients.

Shockable CA-CS patients exhibited significantly higher levels of hepatic cytolysis, with a median ASAT of 189 IU/L [IQR: 87–541] versus 83 IU/L [37–276] in non-CA patients (*p* = 0.003), and a median ALAT of 117 IU/L [53–296] versus 56 IU/L [25–170], respectively (*p* = 0.007) (Table 2). Additionally, hyperlactatemia was more pronounced (median lactate level 4.0 vs. 3.0 mmol/L for shockable CA-CS and non-CA-CS respectively, *p* < 0.001). C-reactive protein levels at admission were significantly lower in shockable CA-CS patients (median: 6 mg/L [IQR: 2–43]) compared to non-CA patients (29 mg/L [10–70]; *p* = 0.003).

**Table 2:** Clinical, echographic, and biological characteristics of cardiogenic shock patients included.

		Overall (*n* = 771)		non-CA-CS (*n* = 692)		CA-CS (*n* = 79)		Age-adj. *p*
First medical contact, *n* (%)								
	Emergency department	257	33.4	252	36.5	5	6.3	(ref)
	Mobile emergency unit	229	29.8	168	24.3	61	77.2	<0.001
	Hospital (already hospitalized)	147	19.1	137	19.9	10	12.7	0.019
	General practitioner	68	8.8	67	9.7	1	1.3	0.846
	Cardiologist	68	8.8	66	9.6	2	2.5	0.472
Admission unit, *n* (%)								<0.001
	CICU	414	70.2	387	74.0	27	40.3	
	Reanimation	176	29.8	136	26.0	40	59.7	
Clinical presentation at admission								
	Heart rate (bpm), mean ± SD	95.6	±29.6	96.1	±29.0	91.4	±34.2	0.071
	*n*	*769*		*691*		*78*		
	SBP (mmHg), mean ± SD	101	±25	101	±25	103	±30	0.728
	*n*	*770*		*691*		*79*		
	DBP (mmHg), mean ± SD	63	±17	63	±17	61	±19	0.143
	*n*	*769*		*691*		*78*		
	Sinus rhythm, *n* (%)	399	52.0	361	52.4	38	48.1	0.238
	Mottling, *n* (%)	256	38.8	221	37.7	35	48.0	0.077
Blood tests at admission								
	Sodium (mmol/l), mean ± SD	135	±6	134	±6	138	±5	<0.001
	*n*	*760*		*683*		*77*		
	eGFR (mL/min/1.73 m^2^), mean ± SD	49.6	±26.6	48.8	±26.5	55.9	±27.4	0.295
	*n*	*751*		*674*		*77*		
	Bilirubin (mg/L), median (IQR)	16 (9–29)		17 (10–31)		12 (8–18)		<0.001^**^
	*n*	*544*		*486*		*58*		
	Haemoglobin (g/dL), mean ± SD	12.5	±2.3	12.5	±2.3	12.6	±2.4	0.861
	*n*	*754*		*678*		*76*		
	Arterial blood lactates (mmol/l), median (IQR)	3.0 (2.0–4.7)		3.0 (2.0–4.0)		4.0 (2.0–7.0)		<0.001^**^
	*n*	*684*		*608*		*76*		
	ASAT (IU/L), median (IQR)	90 (39–301)		83 (37–276)		189 (87–541)		0.003^**^
	*n*	*547*		*493*		*54*		
	ALAT (IU/L), median (IQR)	59 (27–183)		56 (25–170)		117 (53–296)		0.007^**^
	*n*	*559*		*504*		*55*		
	Nt proBNP (pg/mL), median (IQR)	9277 (4052–23256)		9604 (4411–22149)		1309 (724–31347)		0.025^**^
	*n*	*224*		*211*		*13*		
	BNP (pg/mL), median (IQR)	1150 (477–2768)		1352 (602–2844)		210 (75–422)		<0.001*
	*n*	*264*		*239*		*25*		
	CRP (mg/L), median (IQR)	28 (9–69)		29 (10–70)		6 (2–43)		0.003^**^
	*n*	*406*		*381*		*25*		
Baseline echography								
	LVEF (%), mean ± SD	26.3	±13.4	26.1	±13.1	27.6	±15.2	0.141
	*n*	*763*		*685*		*78*		
	TAPSE (mm), mean ± SD	13.4	±5.0	13.2	±4.8	16.0	±6.2	0.015
	*n*	*259*		*239*		*20*		
	PSVtdi (cm/s), median (IQR)	8 (6–11)		8 (6–10)		11 (9–17)		0.001^**^
	*n*	*206*		*190*		*16*		
	Severe mitral regurgitation, *n* (%)	107	14.6	104	15.8	3	4.0	0.010
	Severe aortic stenosis, *n* (%)	36	4.7	34	5.0	2	2.6	0.630
	Severe aortic regurgitation, *n* (%)	10	1.3	8	1.2	2	2.6	0.357

ALAT: ALanine AminoTransferase. ASAT: ASpartate AminoTransferase. CICU: Cardiologic Intensive Care Unit. CRP: C-Reactive Protein. DBP: Diastolic Blood Pressure. IQR: InterQuartile Range. LVEF: Left Ventricular Ejection Fraction. PSVtdi: Peak Systolic Velocity Tissue Doppler Imaging. SBP: Systolic Blood Pressure. SD: Standard Deviation. TAPSE: Tricuspid Annular Plane Systolic Excursion.

### Baseline echocardiographic characteristics

No difference at admission was noticed regarding left ventricular ejection fraction (LVEF) between the two groups. Shockable CA-CS patients also showed a tendency toward less valvular dysfunction, with severe mitral regurgitation present in 4.0 % versus 15.8 % in non-CA patients (*p* = 0.010) (Table 2). The assessment of right ventricular dysfunction through tricuspid annular plane systolic excursion (TAPSE) and tricuspid peak systolic velocity using tissue Doppler imaging was hindered by the substantial amount of missing data in the shockable CA-CS group.

### CS management

Shockable CA-CS patients were more frequently hospitalized from a mobile emergency unit than non-CA-CS patients (77.2 % vs. 24.3 %, *p* < 0.001), consequently, they were also less frequently hospitalized first in the emergency department (6.3 % vs. 36.5 %, *p* < 0.001) or elsewhere in the hospital (12.7 % vs. 19.9 %, *p* < 0.019). As expected, shockable CA-CS patients were more frequently admitted in ICU than in CICU (59.7 % vs. 40.3 %, *p* < 0.001).

### In-hospital management

In hospital management is reported in Table 3. Medical management was different between the two groups, regarding volume expansion, administration of norepinephrine and epinephrine, which was significantly higher in shockable CA-CS patients versus non-CA-CS groups (respectively, 67.5 vs. 39 %; *p* < 0.001, 69.2 % vs. 51.6 %; *p* = 0.003, 37.2 % vs. 9.6 %; *p* < 0.001). Conversely, diuretics were more frequently used in non-CA-CS patients (85.5 vs. 55.1 %, *p* < 0.001), whereas no difference regarding renal replacement therapy was found between the two groups. The administration of dobutamine did not differ significantly between the non-CA-CS group (83 %) and the shockable CA-CS group (75.6 %, *p* = 0.10).

**Table 3:** In-hospital management/discharge.

		Overall (*n* = 771)		non-CA-CS (*n* = 692)		CA-CS (*n* = 79)		Age-adj. *p*
Medications used, *n* (%)								
	Diuretics	633	82.4	590	85.5	43	55.1	<0.001
	Volume expander	321	41.9	269	39.0	52	67.5	<0.001
	Dobutamine	632	82.3	573	83.0	59	75.6	0.083
	If yes, Maximum dose (μg/kg/min):							
	5–10	405	63.8	372	64.7	33	55.0	0.348
	10–15	136	21.4	121	21.0	15	25.0	(ref)
	>15	47	7.4	42	7.3	5	8.3	0.905
	Unknown	47	7.4	40	7.0	7	11.7	0.478
	Norepinephrine	410	53.4	356	51.6	54	69.2	0.006
	If yes, Maximum dose (mg/h)							
	<1	86	21.0	74	20.8	12	22.2	0.737
	1–5	215	52.4	189	53.1	26	48.1	(ref)
	>5	75	18.3	61	17.1	14	25.9	0.151
	Unknown	34	8.3	32	9.0	2	3.7	0.305
	Epinephrine	95	12.4	66	9.6	29	37.2	<0.001
	If yes, Maximum dose (mg/h)							
	<1	34	35.8	23	34.8	11	37.9	(ref)
	1–5	40	42.1	25	37.9	15	51.7	0.690
	>5	14	14.7	11	16.7	3	10.3	0.427
	Unknown	7	7.4	7	10.6	0	0.0	–
	Norepinephrine + dobutamine combination	352	45.8	309	44.8	43	55.1	0.125
	Levosimendan	57	7.4	54	7.8	3	3.9	0.156
	Dopamine	2	0.3	2	0.3	0	0.0	–
	Isoprenaline	32	4.2	26	3.8	6	7.7	0.102
	Antiarrhythmic	298	38.8	271	39.3	27	34.6	0.455
	Transfusion	128	16.7	111	16.1	17	22.1	0.280
	Fibrinolysis	13	1.7	13	1.9	0	0.0	–
Organ replacement therapies, *n* (%)								
	Respiratory support							
	Invasive	291	37.9	229	33.2	62	^2^x	<0.001
	Non invasive	199	25.9	177	25.7	22	28.2	0.555
	Mechanical circulatory support	142	18.5	114	16.5	28	35.4	0.002
	If yes:							
	IABP	48	34.5	40	35.7	8	29.6	0.785
	Impella	26	18.7	24	21.4	2	7.4	0.101
	ECLS	84	60.4	63	56.3	21	77.8	0.061
	Renal replacement therapy	121	15.7	106	15.3	15	19.0	0.564
Invasive cardiology, *n* (%)								
	CAG	398	51.6	338	48.8	60	76.0	<0.001
	If yes:							
	CAG result							
	Normal	74	18.6	67	19.8	7	11.9	(ref)
	1 – Mono	80	20.2	57	16.9	23	39.0	<0.001
	2 – Bi	90	22.7	82	24.3	8	13.6	0.246
	3 – Tri	87	21.9	81	24.0	6	10.2	0.734
	Unknown	66	16.6	51	15.1	15	25.4	0.003
	Culprit lesion	256	80.5	211	79.3	45	86.5	0.331
	Any PCI	217	28.2	175	25.3	42	53.2	<0.001
	Any PCI (even in a second time)	226	29.3	184	26.6	42	53.2	<0.001
	Right heart catheterisation	121	15.8	114	16.5	7	9.0	0.032
	Pace-maker implantation	35	4.8	31	4.7	4	5.2	0.657
	Defibrillator implantation	37	5.1	30	4.6	7	9.1	0.247
	Radiofrequency ablation	17	2.3	16	2.4	1	1.3	0.575
**Discharge**								
	LVEF (%), mean ± SD	35.0	±14.5	34.1	±14.4	42.5	±12.6	0.002
	*n*	*439*		*396*		*43*		
	LVEF variation*, mean ± SD	+8.5	±14.2	+7.9	±13.9	+13.3	±16.0	0.130
	*n*	*436*		*393*		*43*		
	Length of stay in CICU (days), median (IQR)	11(7-21)		12(7-21)		10(5-16)		0.044**
	*n*	*440*		*395*		*45*		
	Length of stay in hospital (days), median (IQR)	17(11-28)		17(11-28)		17(11-27)		0.417**
	*n*	*436*		*393*		*43*		
	Discharge mode							
	Home	171	26.4	153	26.4	18	26.1	(ref)
	Rehabilitation	44	6.8	36	6.2	8	11.6	0.096
	Transferred (other center/other department)	214	33.0	196	33.9	18	26.1	0.519
	Death	216	33.3	191	33.0	25	36.2	0.473
	Other	3	0.5	3	0.5	0	0.0	–
	Registration on transplant waiting list	40	6.4	37	6.7	3	4.5	0.187

* At discharge compared with admission.

** Using log-transformed variable.

Another major difference was the use of respiratory support, which was much more frequent in shockable CA-CS patients (78.5 % vs. 33.2 %, *p* < 0.001). Mechanical circulatory support was also much more frequent in shockable CA-CS patients (35.4 % vs. 16.5 %, *p* < 0.001). There was more coronary angiography performed in shockable CA-CS patients (76 % vs. 48.8 %, *p* < 0.001), highlighting more mono-troncular lesions (39 % vs. 16.9 %, *p* < 0.001) and leading to more percutaneous coronary intervention than in non-CA-CS patients (53.2 % vs. 25.3 %, *p* < 0.001).

There was no difference between groups regarding the median length of stay in ICU and/or CICU or discharge mode after hospitalization. At discharge, left ventricular ejection fraction was higher in shockable CA-CS patients compared to non-CA-CS patients (42.5 % vs. 34.1 %, *p* < 0.001).

### Thirty-day and one-year outcomes and correlates

**Fig. 1:**
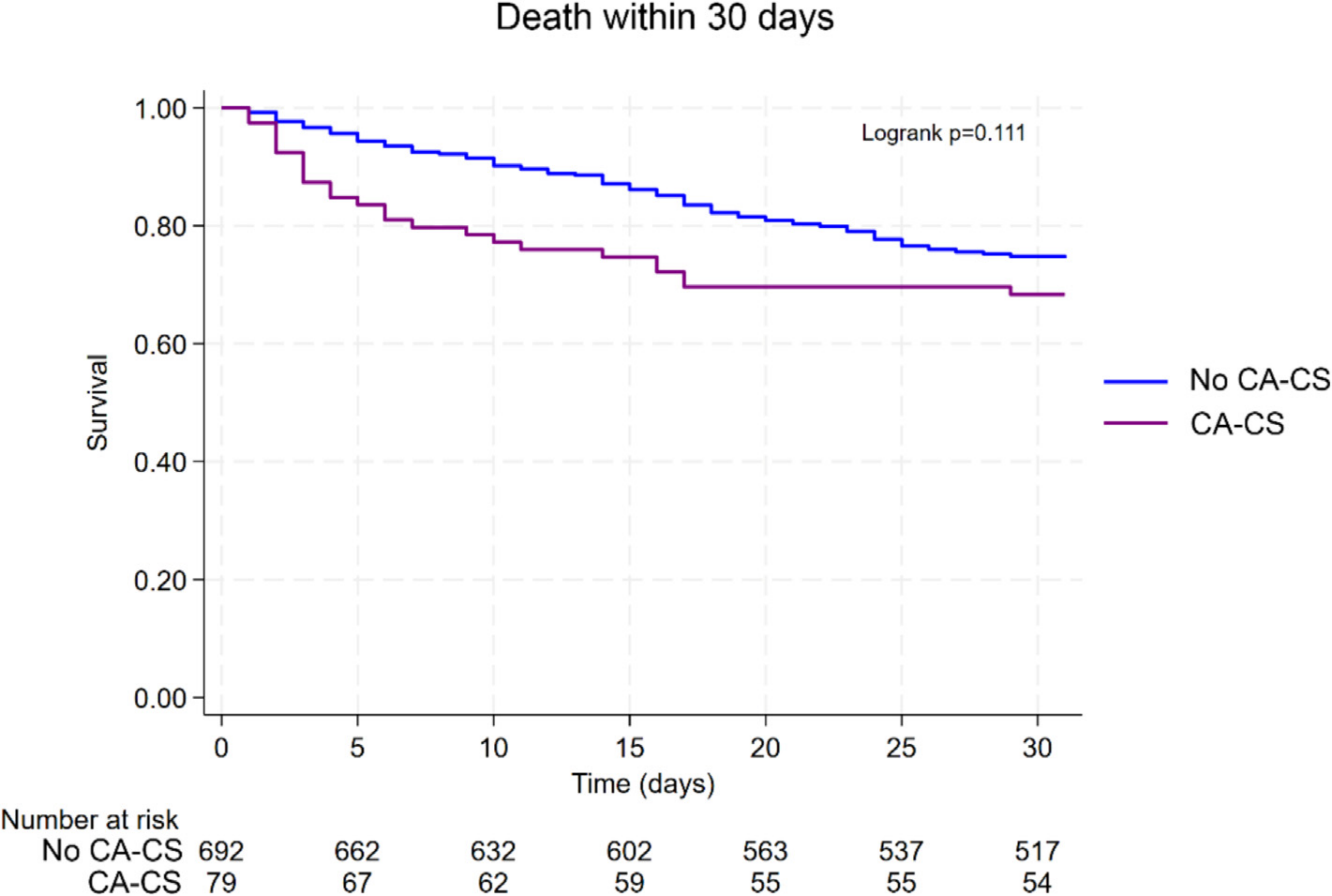
Early (30-day) mortality in patients with and without prior resuscitated shockable CA before the onset of CS. Kaplan-Meier Landmark analysis of failure rate (all-cause death) of 30-day survivors. CA-CS: CS following resuscitated shockable CA. No CA-CS: CS without prior resuscitated CA.

**Fig. 2:**
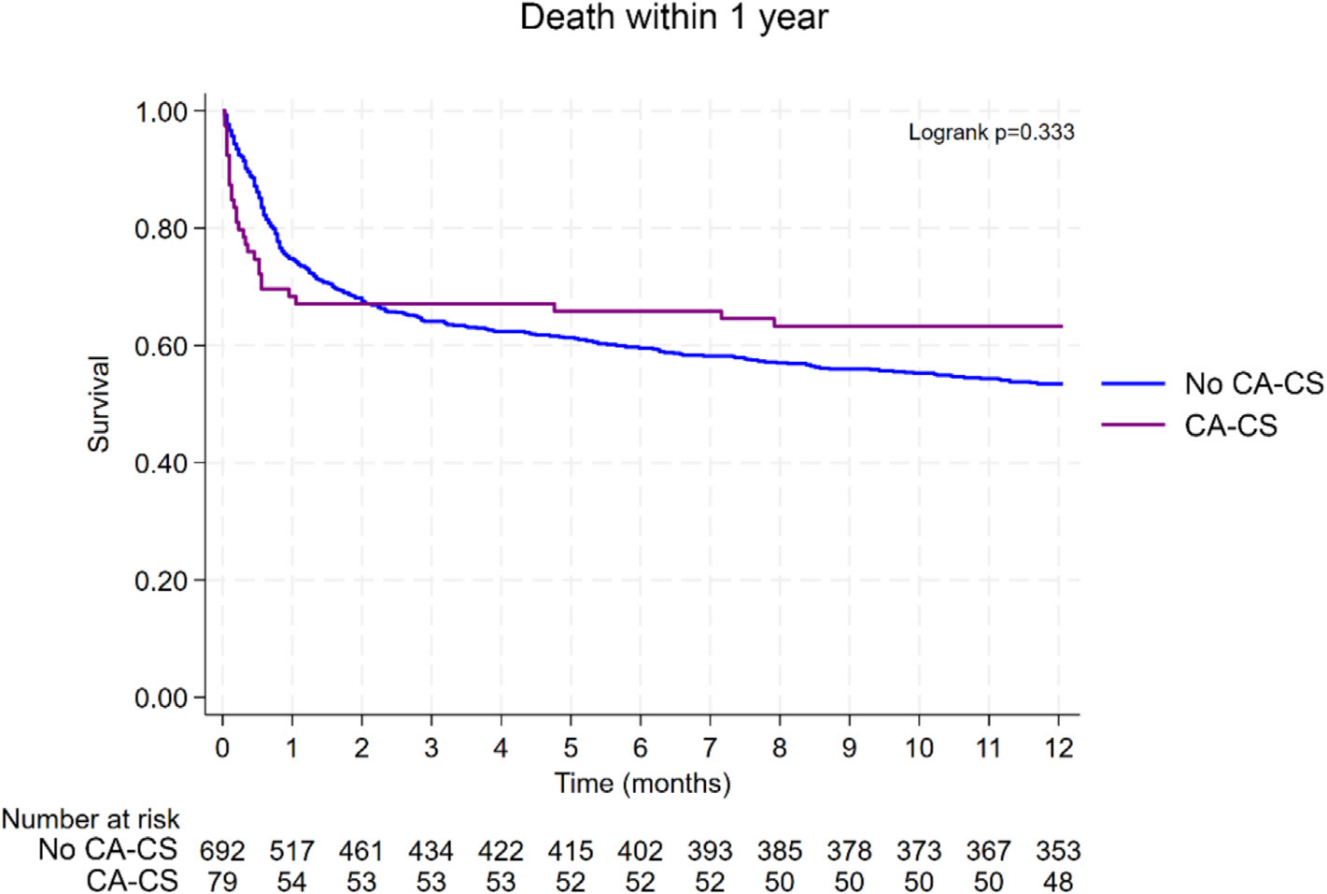
Long-term (1-year) mortality in patients with and without prior resuscitated shockable CA before the onset of CS CA-CS: CS following resuscitated shockable CA. No CA-CS: CS without prior resuscitated CA.

**Fig. 3:**
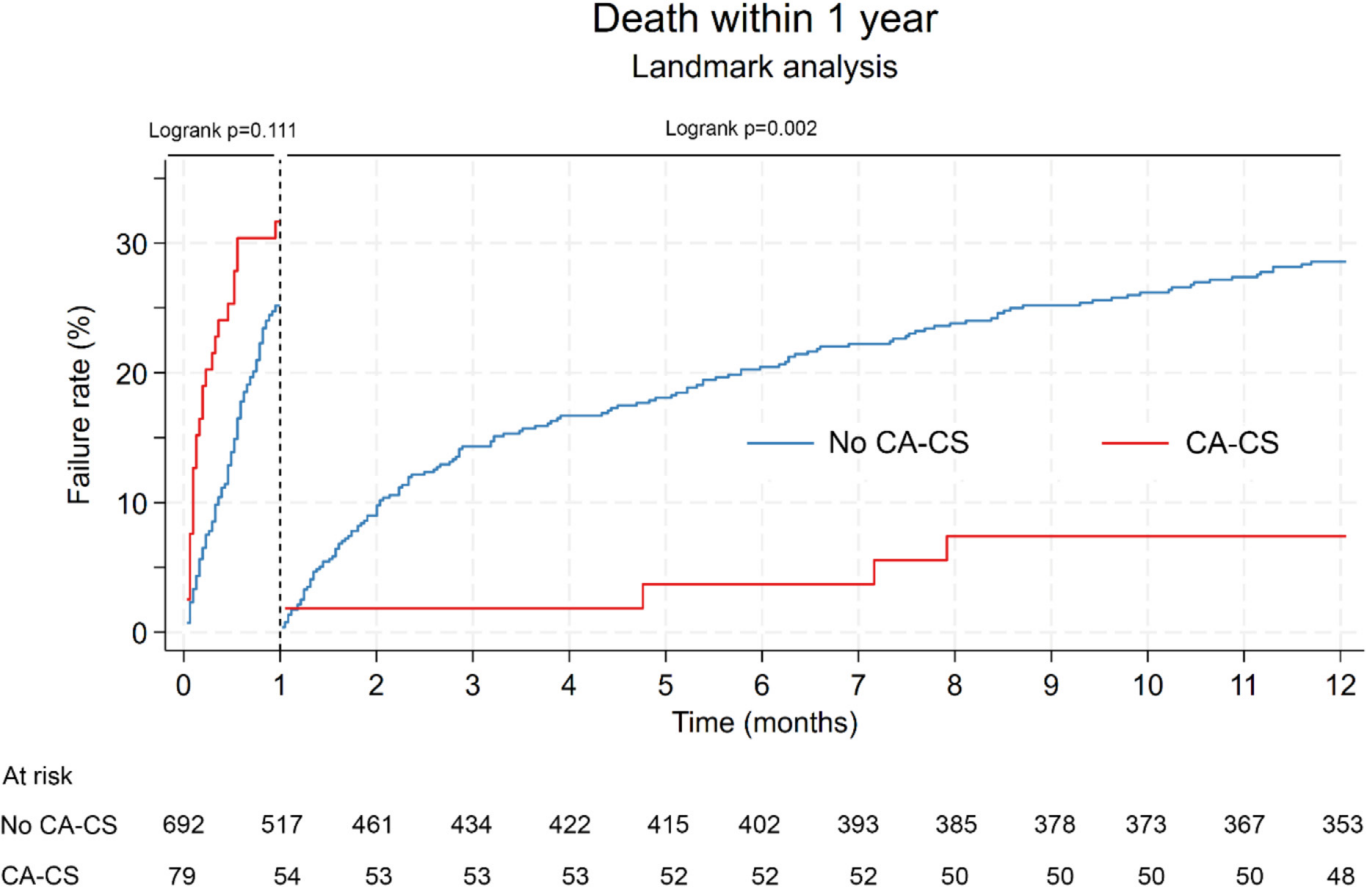
Kaplan-Meier Landmark analysis of failure rate (all-cause death) of 30-day survivors in patients with and without prior resuscitated shockable CA before the onset of CS. Kaplan-Meier Landmark analysis of failure rate (all-cause death) among 30-day survivors. CA-CS: CS following resuscitated shockable CA. No CA-CS: CS without prior resuscitated CA.

The mortality rate at 30 days was similar in shockable CA-CS patients compared to non-CA-CS patients (31.7 % vs. 25.4 %, Logrank *p* = 0.11) (Fig. 1). The mortality rate at one year was not statistically different, with 36.7 % mortality in the shockable CA-CS population and 46.1 % in the non-CA-CS population (Logrank *p* = 0.33) (Fig. 2). However, among 30-day survivors, shockable CA-CS exhibited significantly better long-term survival than the non-CA-CS population (Logrank *p* = 0.002) (Fig. 3).

Patients in the shockable CA-CS group had significantly higher LVEF at discharge (42.5 % ± 12.6 %) compared to the non-CA-CS group (34.1 % ± 14.4 %, p < 0.001). This followed a significant increase in LVEF of 13.3 % (SD = 16) in the shockable CA-CS population, whereas the non-CA-CS population only experienced a 7.9 % (SD = 13.9) improvement (*P* = 0.018).

Age-adjusted univariate analysis was performed to identify baseline characteristics associated with 30-day and 1-year mortality in both populations (Table S1). Severe aortic stenosis and a clinical profile of congestion (marked by elevated natriuretic peptide levels and routine furosemide treatment) were identified as significant key factors associated with increased 30-day and 1-year mortality in shockable CA-CS patients.

In shockable CA-CS population, the multivariable analysis identified 4 independent factors at admission associated with higher mortality at 30 days: age (aHR = 1.03 [1.01–1.06]), previous treatment by furosemide (aHR = 4.04 [1.66–9.83]); CS trigger classified as “other” (aHR = 4.1 [1.31–12.79]) and severe aortic stenosis (aHR = 10.28 [2.09–50.48]) (Table 4).

**Table 4:** Stratified multivariate analysis of baseline characteristics associated with 30-day mortality in shockable CA-CS and non-CA-CS populations.

Death within 30 days – Multivariate Cox Regression							
		CA-CS (*n* = 79)			non-CA-CS (*n* = 692)		
		HR	95 % CI	*p*	HR	95 % CI	*p*
Age (years)		1.03	1.01–1.06	0.021	1.03	1.02–1.04	<0.001
Chronic renal failure					1.50	1.07–2.10	0.018
Previous treatment: Furosemide		4.04	1.66–9.83	0.002			
Trigger: infectious					1.99	1.38–2.86	<0.001
Trigger: other		4.10	1.31–12.79	0.015			
SBP (for 10 mmHg increase)					0.91	0.85–0.97	0.005
Arterial blood lactates (mmol/l)							
	Tertile 1				1.00	(ref)	
	Tertile 2				1.40	0.96–2.04	0.085
	Tertile 3				1.81	1.21–2.70	0.004
	*Unknown value*				*0.92*	*0.52*–*1.65*	*0.791*
LVEF (for 10 % increase)					0.85	0.75–0.96	0.009
Severe aortic stenosis		10.28	2.09–50.48	0.004			

In non-CA-CS population, the multivariable analysis identified 3 independent factors at admission associated with higher mortality at 30 days: age (aHR = 1.03 [1.02–1.04]), chronic renal failure (aHR = 1.5 [1.07–2.1]); CS trigger classified as “infectious” (aHR = 1.99 [1.38–1-2.86]), tertile 2 and 3 of the arterial blood lactate (aHR = 1.4 [0.96–2.4] and 1.81 [1.21–2.70]). Conversely, among the non-CA-CS population, a higher SBP and LVEF at admission were independent factors associated with lower mortality at 30 days (Table 4).

## Discussion

The present study, conducted within the FRENSHOCK registry, aimed to elucidate the clinical outcomes of patients who developed CS following a resuscitated CA with a shockable rhythm compared to those with CS arising from other etiologies (non-CA-CS).

While the annual incidence of cardiogenic shock (CS) in the U.S. adult population is approximately 15 per 100,000^18^, the incidence of out-of-hospital cardiac arrest (OHCA) treated by emergency medical services is significantly higher—ranging from 66 to 82 per 100,000 adults per year^19^. Notably, fewer than 20 % of these OHCA patients present with an initial shockable rhythm.^20^ Indeed, our results about a scarcely, but of utmost importance, issue in CS show that shockable CA-CS patients have similar 30-day and one-year outcomes compared to patients with CS secondary to other causes (non-CA-CS). Even though they usually present at admission with different ages, medical history, and triggers of CS, but also clinical, biological, and echocardiographic differences.

Our results are in line with those of other studies, which found no significant differences between CS patients with and without prior resuscitated CA. Indeed, in a retrospective study by Ostenfeld et al., the authors examined the effects of OHCA on outcomes in patients with acute myocardial infarction (AMI) complicated by CS.^21^ The study revealed that among 248 consecutive patients admitted alive to a tertiary center with the diagnosis of AMI-CS, 48 % presented with OHCA and 52 % without. Although these patients had higher lactate levels upon admission, out-of-hospital CA was not found to be an independent predictor of mortality. Moreover, a large Danish cohort study (*n* = 1,716) including patients with AMICS admitted to two tertiary heart centers (2010–2017) was conducted, capturing approximately two-thirds of the Danish AMICS population.^22^ Interestingly, patients with and without prior OHCA initially presented with similar metabolic and hemodynamic profiles. However, during the first days in ICU, marked differences emerged between the groups. Despite unadjusted analysis suggesting no significant difference in 30-day mortality between OHCA and non-OHCA patients, the cause of in-hospital death differed substantially. Hypoxic brain injury, leading to withdrawal of life support, was the primary cause of death in the OHCA group (56 %), compared to only 4 % in the non-OHCA group. Conversely, CS was the leading cause of death in the non-OHCA group (60 %) compared to 27 % in the OHCA group.^22^ Similarly, in a post hoc study of the HYPO-ECMO trial,^7^ there was no significant difference between CS patients with or without prior resuscitated CA supported by venoarterial extracorporeal membrane oxygenation.^23^ In this study, however, it is important to note that patients with cardiopulmonary resuscitation exceeding 45 min and out-of-hospital refractory CA were excluded.

Our observation is notable given the substantial physiological and metabolic derangements associated with CA, which can often lead to prolonged periods of ischemia and reperfusion injury, leading to multiple organ failure and ischemic encephalopathy.^24^ It is possible that the rigorous management strategies employed in contemporary critical care, including advanced mechanical circulatory support and targeted therapies, have contributed to mitigating the adverse effects of prior CA on outcomes in patients with CS.^25^

Our study focused exclusively on patients who developed CS following a CA and did not consider the reverse scenario, where a CA occurred secondary to pre-existing ongoing CS during hospitalization. This distinction is crucial, as the underlying pathophysiology and prognostic implications of these two scenarios differ, as it was recently evaluated in a retrospective, single-center study by Ahmed et al., who investigated the incidence, clinical characteristics, and outcomes of in-hospital CA in patients admitted to the CICU for CS. Among 1,498 patients with CS, 34 % experienced CA during their hospitalization in CICU, with ventricular fibrillation as the most common trigger (50.6 %). In this study, patients with in-hospital CA during CS management experienced significantly higher rates of both in-hospital (51 % vs. 24.2 %, *p* < 0.001) and 1-year mortality (adjusted hazard ratio 1.53, *p* < 0.001).^26^

Interestingly, one of the findings of our study was that among survivors beyond 30 days, patients with shockable CA-CS exhibited better long-term survival compared to those with non-CA-CS. This may be explained by differences in baseline characteristics (shockable CA-CS patients were younger with less history of cardiac disease) and by the greater improvement in LVEF observed at discharge in the shockable CA-CS group. These results underscore the importance of avoiding therapeutic nihilism in carefully selected CA-CS patients, as a more aggressive management approach could potentially yield favorable outcomes. To better identify patients at risk of unfavorable outcomes in CA-CS, a recent study by Besch et al. highlighted several key predictors of poorer prognosis, including prolonged cardiopulmonary resuscitation (CPR), unwitnessed CA, and OHCA, all of which are strongly associated with an increased risk of anoxic brain injury.^27^

However, it is essential to acknowledge the limitations of our study. As in any observational study, there are limitations to our analysis. First, patients were enrolled from ICU and CICU (directly or after transfer from another center), and we cannot exclude the possibility that severe comorbid, older, or most severe cases with multiple organ failure could have not been transferred for futility. Moreover, data for patients who died early (before informed consent was obtained) were not collected and recorded in the database because of administrative regulations. Thus, a potential 'healthy survivor bias' may have contributed to an underestimation of mortality, particularly in the shockable CA-CS group.^28^ Patients with shockable CA-CS who did not survive long enough for ICU admission or those who died early before informed consent could be obtained were excluded, likely leading to a selection of healthier individuals within this group. This selection bias may help explain the absence of excess mortality, despite the more severe clinical presentation in shockable CA-CS patients. Nonetheless, the younger age of shockable CA-CS patients likely played a significant role in their improved survival between 30 days and 1 year. Additionally, regarding patients with CA who were included, the incompleteness of Utstein criteria^29^ data limits the generalizability of our findings. Indeed, information on prognostic modifiers main factors, such as the initial location of CA and no-flow or low-flow durations, proportion of witnessed and bystander CPR, and the cause-of-death data, was not available. One limitation of our analysis is the use of backward selection, which may omit true confounders or introduce bias through over-adjustment or collider effects. Another limitation to mention is that SCAI SHOCK Stage Classification was not used for the CS severity classification, given that this score was not yet available at the time of the study, since it was first published in 2019.^30^ To our knowledge, this is the first study to comprehensively address features and outcomes of CS resulting from diverse etiologies (including ischemic and non-ischemic) in patients with and without prior resuscitated CA.

In accordance with expert recommendations, CA should be more precisely defined within the context of CS studies and trials, to include only cases that do not inherently predispose to more severe outcomes than the general CS population.^4^

## Conclusion

In a cohort of patients with cardiogenic shock from various etiologies, approximately 10 % had experienced prior resuscitation following a cardiac arrest with shockable rhythms. Our findings suggest that while a resuscitated cardiac arrest with a shockable rhythm is a recognized risk factor for the development of cardiogenic shock, selected cardiac arrest with a shockable rhythm leading to cardiogenic shock does not inherently confer a worse prognosis compared to other causes of cardiogenic shock. However, further research is needed to fully elucidate the long-term outcomes in this population and to determine the appropriateness of including patients with cardiogenic shock secondary to cardiac arrest in future clinical trials.

## Consent for publication

All authors hereby consent to the publication.

## Availability of data and material

All summarized data are available upon request.

## CRediT authorship contribution statement

**Hamid Merdji:** Writing – review & editing, Writing – original draft, Formal analysis, Conceptualization. **Vincent Bataille:** Writing – review & editing, Data curation. **Anais Curtiaud:** Writing – review & editing. **Laurent Bonello:** Writing – review & editing. **François Roubille:** Writing – review & editing. **Bruno Levy:** Writing – review & editing. **Pascal Lim:** Writing – review & editing. **Jean-Claude Dib:** Writing – review & editing. **Julien Maizel:** Writing – review & editing. **Nicolas Brechot:** Writing – review & editing. **Marion Beuzelin:** Writing – review & editing. **Emmanuelle Fillippi:** Writing – review & editing. **Miloud Cherbi:** Writing – review & editing. **Julien Demiselle:** Writing – review & editing. **Grégoire Rangé:** Writing – review & editing. **Jérémie Joffre:** Writing – review & editing. **Marwan Yassine:** Writing – review & editing. **Caroline Biendel:** Writing – review & editing. **Fanny Bounes:** Writing – review & editing. **Guillaume Leurent:** Writing – review & editing. **Edouard Gerbaud:** Writing – review & editing. **Eric Bonnefoy:** Writing – review & editing. **Etienne Puymirat:** Writing – review & editing. **Clément Delmas:** Writing – review & editing. **Nadia Aissaoui:** . **François Bagate:** . **Florence Boissier:** . **Éric Bonnefoy-Cudraz:** . **Marie Boughenou:** . **Stéphane Boule:** . **Jérémie Bourenne:** . **Cédric Bruel:** . **Alain Cariou:** . **Philippe Castellant:** . **Sébastien Champion:** . **Karim Chaoui:** . **Marion Chatot:** . **Nicolas Combaret:** . **Nicolas Debry:** . **Xavier Delabranche:** . **Raphael Favory:** . **Emmanuelle Filippi:** . **Romain Gallet:** . **Frédérique Ganster:** . **Philippe Gaudard:** . **Brahim Harbaoui:** . **Patrick Henry:** . **Benoit Herce:** . **Fabrice Ivanes:** . **Philippe Karoubi:** . **Hadi Khachab:** . **Khalifé Khalife:** . **Kada Klouche:** . **Vincent Labbe:** . **Marc Laine:** . **Nicolas Lamblin:** . **Benoit Lattuca:** . **Yann Lefetz:** . **Gilles Lemesle:** . **Philippe Letocart:** . **Leurent:** . **Guillaume Louis:** . **Jacques Mansourati:** . **Stéphane Manzo-Silberman:** . **Séverine Marchand:** . **Benjamin Marchandot:** . **Stéphanie Marliere:** . **Joy Mootien:** . **Frédéric Mouquet:** . **Louis Niquet:** . **Alexis Paternot:** . **Vincent Probst:** . **Charlotte Quentin:** . **Grégoire Range:** . **Nassim Redjimi:** . **Jean Christophe Richard:** . **Christophe Saint Etienne:** . **Francis Schneider:** . **Guillaume Schurtz:** . **Marie-France Seronde:** . **Julien Ternacle:** . **Gérald Vanzetto:** . **Elie Zogheib:** .

## Ethics approval and consent to participate

The data recorded, their handling, and storage were reviewed and approved by the CCTIRS (French Health Research Data Processing Advisory Committee) (no. 15.897) and the CNIL (French Data Protection Agency) (no. DR-2016-109).

## Funding

The study was sponsored by the Fédération Française de Cardiologie and was funded by unrestricted grants from Daiichi-Sankyo and Maquet SAS.

## Declaration of competing interest

The authors declare that they have no known competing financial interests or personal relationships that could have appeared to influence the work reported in this paper.

## Appendix A Supplementary material

Supplementary STROBE checklist
