# Supplementary material for: Comparative features and outcomes of cardiogenic shock in patients with and without prior resuscitated shockable cardiac arrest: Insight from the FRENSHOCK multicenter prospective registry
Source: Resusc Plus. 2025 Jul 9;25:101024. doi: 10.1016/j.resplu.2025.101024 (PMC12312061; doi:10.1016/j.resplu.2025.101024)
Supplement: Supplementary Table S1 [file mmc2.docx]

**Table S1: Age-adjusted univariate analysis of baseline characteristics associated with 30-day and 1-year mortality in shockable CA-CS and non-CA-CS population.**

|  |  |  | **Death within 30 days** | | | | | | **Death within 1 year** | | | | | |
| --- | --- | --- | --- | --- | --- | --- | --- | --- | --- | --- | --- | --- | --- | --- |
|  |  |  | **Cardiac arrest (n=79)** | | | **No cardiac arrest (n=692)** | | | **Cardiac arrest (n=79)** | | | **No cardiac arrest (n=692)** | | |
|  |  |  | **Age-adj.HR** | **95% CI** | **p** | **Age-adj.HR** | **95% CI** | **p** | **Age-adj.HR** | **95% CI** | **p** | **Age-adj.HR** | **95% CI** | **p** |
|  | **Characteristics at admission** | |  |  |  |  |  |  |  |  |  |  |  |  |
|  | Male gender | | 1,64 | 0,38 - 7,16 | 0,509 | 1,05 | 0,76 - 1,45 | 0,769 | 2,10 | 0,49 - 9,03 | 0,321 | 0,96 | 0,76 - 1,21 | 0,713 |
|  | Age (years) | | **1,04** | **1,01 - 1,06** | **0,008** | **1,03** | **1,02 - 1,04** | **< 0,001** | **1,03** | **1,01 - 1,06** | **0,012** | **1,03** | **1,02 - 1,04** | **< 0,001** |
|  | BMI (kg/m²) | | 0,97 | 0,87 - 1,07 | 0,518 | 1,00 | 0,97 - 1,03 | 0,877 | 0,99 | 0,90 - 1,08 | 0,759 | 0,99 | 0,97 - 1,01 | 0,454 |
|  | Risk factors | |  |  |  |  |  |  |  |  |  |  |  |  |
|  |  | Current smoker | **2,49** | **1,02 - 6,07** | **0,045** | 0,97 | 0,65 - 1,45 | 0,895 | **2,32** | **1,02 - 5,27** | **0,045** | 0,96 | 0,72 - 1,28 | 0,783 |
|  |  | Diabetes mellitus | 1,27 | 0,51 - 3,20 | 0,606 | 0,75 | 0,54 - 1,06 | 0,101 | 1,83 | 0,83 - 4,02 | 0,133 | 0,92 | 0,72 - 1,17 | 0,499 |
|  |  | Arterial hypertension | 0,87 | 0,37 - 2,02 | 0,741 | 0,81 | 0,59 - 1,12 | 0,204 | 0,77 | 0,35 - 1,68 | 0,508 | 0,88 | 0,69 - 1,11 | 0,272 |
|  |  | Dyslipidaemia | 0,72 | 0,30 - 1,72 | 0,456 | 0,87 | 0,64 - 1,18 | 0,360 | 0,84 | 0,38 - 1,84 | 0,656 | 1,12 | 0,89 - 1,40 | 0,326 |
|  | Medical history | |  |  |  |  |  |  |  |  |  |  |  |  |
|  |  | History of cardiac disease | 1,67 | 0,73 - 3,85 | 0,226 | 1,01 | 0,75 - 1,37 | 0,942 | 1,79 | 0,82 - 3,87 | 0,143 | **1,43** | **1,13 - 1,80** | **0,003** |
|  |  | Ischaemic | 1,77 | 0,78 - 4,01 | 0,173 | 0,77 | 0,55 - 1,07 | 0,115 | 1,72 | 0,80 - 3,72 | 0,164 | 1,08 | 0,86 - 1,37 | 0,498 |
|  |  | Hypertrophic | *not computed* | | | 0,79 | 0,20 - 3,20 | 0,745 | *not computed* | | | 0,83 | 0,31 - 2,24 | 0,719 |
|  |  | Idiopathic | 1,07 | 0,25 - 4,58 | 0,924 | 1,16 | 0,73 - 1,85 | 0,536 | 0,96 | 0,23 - 4,05 | 0,953 | 1,16 | 0,82 - 1,66 | 0,398 |
|  |  | Toxic | *not computed* | | | 0,81 | 0,36 - 1,84 | 0,621 | *not computed* | | | 0,98 | 0,57 - 1,69 | 0,950 |
|  |  | Multisite pacing | 3,71 | 0,48 - 28,75 | 0,210 | 0,91 | 0,55 - 1,50 | 0,705 | 3,23 | 0,42 - 24,80 | 0,259 | 1,06 | 0,74 - 1,52 | 0,736 |
|  |  | Defibrillator | 1,72 | 0,23 - 12,87 | 0,597 | 0,97 | 0,66 - 1,45 | 0,893 | 1,50 | 0,20 - 11,11 | 0,692 | 1,26 | 0,96 - 1,66 | 0,100 |
|  |  | CABG | 2,23 | 0,67 - 7,47 | 0,193 | 0,91 | 0,54 - 1,52 | 0,719 | 2,77 | 0,96 - 8,00 | 0,059 | 1,12 | 0,77 - 1,61 | 0,556 |
|  |  | PCI | 1,06 | 0,42 - 2,68 | 0,896 | 0,79 | 0,55 - 1,14 | 0,214 | 0,91 | 0,37 - 2,24 | 0,829 | 1,08 | 0,84 - 1,40 | 0,540 |
|  |  | Peripheral artery disease | *not computed* | | | 0,75 | 0,46 - 1,20 | 0,229 | 0,31 | 0,04 - 2,27 | 0,249 | 0,87 | 0,62 - 1,21 | 0,409 |
|  |  | Ischemic stroke | 1,80 | 0,61 - 5,34 | 0,287 | 1,13 | 0,68 - 1,89 | 0,640 | 2,09 | 0,78 - 5,62 | 0,142 | 1,41 | 0,98 - 2,03 | 0,060 |
|  |  | Chronic renal failure | 1,05 | 0,35 - 3,15 | 0,927 | **1,42** | **1,02 - 1,97** | **0,036** | 1,24 | 0,46 - 3,35 | 0,667 | **1,47** | **1,15 - 1,89** | **0,002** |
|  |  | Dialysis | *not computed* | | | 1,28 | 0,41 - 4,02 | 0,667 | *not computed* | | | 1,13 | 0,47 - 2,73 | 0,791 |
|  |  | COPD | *not computed* | | | 1,37 | 0,83 - 2,26 | 0,221 | 0,82 | 0,11 - 6,07 | 0,846 | 1,38 | 0,95 - 2,01 | 0,096 |
|  |  | Cancer | 0,71 | 0,10 - 5,30 | 0,740 | 1,17 | 0,68 - 2,02 | 0,577 | 1,32 | 0,31 - 5,61 | 0,707 | **1,75** | **1,23 - 2,50** | **0,002** |
|  | Previous medications | |  |  |  |  |  |  |  |  |  |  |  |  |
|  |  | Aspirin | 1,25 | 0,57 - 2,77 | 0,579 | 0,93 | 0,68 - 1,27 | 0,654 | 1,23 | 0,59 - 2,56 | 0,587 | 0,96 | 0,77 - 1,21 | 0,742 |
|  |  | P2Y12 inhibitor | 1,25 | 0,47 - 3,32 | 0,661 | 0,78 | 0,51 - 1,20 | 0,258 | 1,61 | 0,69 - 3,78 | 0,271 | 0,81 | 0,59 - 1,10 | 0,174 |
|  |  | Statins | 1,11 | 0,49 - 2,50 | 0,803 | 0,86 | 0,63 - 1,17 | 0,329 | 1,05 | 0,49 - 2,27 | 0,891 | 0,91 | 0,73 - 1,15 | 0,443 |
|  |  | Betablockers | 1,02 | 0,46 - 2,34 | 0,958 | 0,94 | 0,70 - 1,28 | 0,711 | 0,84 | 0,38 - 1,85 | 0,660 | 1,11 | 0,89 - 1,38 | 0,366 |
|  |  | Vitamin K antagonist | 1,48 | 0,47 - 4,66 | 0,507 | 1,28 | 0,92 - 1,77 | 0,138 | 1,40 | 0,45 - 4,33 | 0,561 | **1,39** | **1,09 - 1,77** | **0,008** |
|  |  | Direct oral anticoagulant | 4,81 | 0,63 - 36,61 | 0,130 | **0,44** | **0,21 - 0,93** | **0,032** | 4,76 | 0,63 - 36,19 | 0,132 | 0,70 | 0,45 - 1,09 | 0,113 |
|  |  | ACE inhibitors or ARB | 0,75 | 0,32 - 1,78 | 0,520 | 1,00 | 0,74 - 1,35 | 0,997 | 0,63 | 0,27 - 1,44 | 0,272 | 1,03 | 0,83 - 1,29 | 0,778 |
|  |  | Sacubitril / valsartan | *not computed* | | | 0,62 | 0,20 - 1,95 | 0,418 | *not computed* | | | 1,67 | 0,93 - 2,97 | 0,084 |
|  |  | Furosemide | 2,87 | 1,28 - 6,45 | 0,011 | 1,13 | 0,84 - 1,53 | 0,411 | **3,21** | **1,52 - 6,79** | **0,002** | **1,46** | **1,17 - 1,83** | **0,001** |
|  |  | Aldosterone antagonist | *not computed* | | | 1,02 | 0,67 - 1,55 | 0,924 | *not computed* | | | 1,22 | 0,91 - 1,65 | 0,182 |
|  |  | Amiodarone | 1,56 | 0,45 - 5,34 | 0,483 | 0,98 | 0,66 - 1,45 | 0,917 | 1,85 | 0,62 - 5,47 | 0,267 | 1,26 | 0,96 - 1,65 | 0,091 |
|  |  | Proton pump inhibitor | 0,70 | 0,26 - 1,90 | 0,485 | 0,90 | 0,66 - 1,23 | 0,502 | 0,92 | 0,39 - 2,20 | 0,857 | 1,18 | 0,94 - 1,48 | 0,149 |
|  | Triggers | |  |  |  |  |  |  |  |  |  |  |  |  |
|  |  | Ischaemic | 1,23 | 0,54 - 2,79 | 0,628 | 0,97 | 0,71 - 1,32 | 0,858 | 1,32 | 0,61 - 2,84 | 0,478 | 0,86 | 0,68 - 1,08 | 0,201 |
|  |  | Mechanical | *not computed* | | | 1,63 | 0,83 - 3,19 | 0,154 | *not computed* | | | 1,32 | 0,76 - 2,30 | 0,330 |
|  |  | Ventricular arrhythmia | 1,38 | 0,61 - 3,12 | 0,444 | 0,97 | 0,59 - 1,59 | 0,893 | 1,43 | 0,67 - 3,05 | 0,348 | 0,95 | 0,65 - 1,37 | 0,780 |
|  |  | Atrial arrhythmia | 1,04 | 0,31 - 3,49 | 0,946 | 0,91 | 0,59 - 1,41 | 0,677 | 0,91 | 0,27 - 2,99 | 0,870 | 0,93 | 0,67 - 1,28 | 0,660 |
|  |  | Conductive disorders | 1,11 | 0,25 - 4,90 | 0,889 | 0,24 | 0,03 - 1,72 | 0,157 | 1,02 | 0,23 - 4,43 | 0,984 | 0,83 | 0,39 - 1,76 | 0,629 |
|  |  | Infectious | 0,72 | 0,08 - 6,31 | 0,766 | **2,17** | **1,52 - 3,09** | **< 0,001** | 0,69 | 0,08 - 5,85 | 0,731 | 1,94 | 1,46 - 2,58 | < 0,001 |
|  |  | Non compliance | *not computed* | | | 1,31 | 0,67 - 2,57 | 0,425 | *not computed* | | | 1,57 | 0,98 - 2,53 | 0,063 |
|  |  | Iatrogenic | *not computed* | | | 0,71 | 0,38 - 1,36 | 0,305 | 3,11 | 0,41 - 23,33 | 0,270 | 0,85 | 0,55 - 1,31 | 0,458 |
|  |  | Other | 2,48 | 0,85 - 7,25 | 0,098 | 0,63 | 0,37 - 1,07 | 0,084 | 2,15 | 0,74 - 6,22 | 0,157 | 0,80 | 0,56 - 1,13 | 0,206 |
|  |  | None / undefined | 2,39 | 0,71 - 8,10 | 0,160 | 1,10 | 0,75 - 1,63 | 0,623 | 2,15 | 0,64 - 7,17 | 0,214 | 1,08 | 0,80 - 1,45 | 0,621 |
|  | **Clinical, echographic and biological presentation** | |  |  |  |  |  |  |  |  |  |  |  |  |
|  | Admission unit | |  |  |  |  |  |  |  |  |  |  |  |  |
|  |  | CICU | 1,00 | (ref) |  | 1,00 | (ref) |  | 1,00 | (ref) |  | 1,00 | (ref) |  |
|  |  | Reanimation | 1,85 | 0,71 - 4,83 | 0,207 | **1,56** | **1,08 - 2,26** | **0,018** | 1,33 | 0,58 - 3,04 | 0,502 | **1,40** | **1,06 - 1,85** | **0,017** |
|  | Clinical presentation at admission | |  |  |  |  |  |  |  |  |  |  |  |  |
|  |  | Heart rate (for 10 bpm) | 1,05 | 0,94 - 1,16 | 0,394 | **1,05** | **1,001 - 1,10** | **0,047** | 1,02 | 0,93 - 1,13 | 0,654 | 1,02 | 0,98 - 1,06 | 0,315 |
|  |  | SBP (for 10 mmHg) | 0,96 | 0,84 - 1,09 | 0,522 | **0,90** | **0,84 - 0,96** | **0,002** | 0,95 | 0,84 - 1,08 | 0,431 | **0,92** | **0,88 - 0,96** | **< 0,001** |
|  |  | DBP (for 10 mmHg) | 0,87 | 0,68 - 1,12 | 0,273 | **0,89** | **0,81 - 0,97** | **0,011** | 0,82 | 0,65 - 1,04 | 0,095 | **0,88** | **0,82 - 0,94** | **< 0,001** |
|  |  | Sinus rhythm | 0,51 | 0,22 - 1,14 | 0,102 | 0,88 | 0,65 - 1,18 | 0,384 | 0,54 | 0,25 - 1,14 | 0,106 | 0,90 | 0,72 - 1,13 | 0,359 |
|  |  | Mottling | 1,04 | 0,45 - 2,39 | 0,925 | **1,39** | **1,01 - 1,93** | **0,044** | 1,07 | 0,50 - 2,30 | 0,861 | **1,44** | **1,14 - 1,82** | **0,002** |
|  | Blood tests at admission | |  |  |  |  |  |  |  |  |  |  |  |  |
|  |  | Sodium (mmol/l) | 0,97 | 0,88 - 1,06 | 0,474 | 0,98 | 0,96 - 1,00 | 0,092 | 0,95 | 0,87 - 1,04 | 0,279 | **0,97** | **0,95 - 0,99** | **0,004** |
|  |  | eGFR (for 10 mL/min/1.73 m²) | 0,84 | 0,70 - 1,01 | 0,067 | **0,89** | **0,83 - 0,95** | **0,001** | **0,83** | **0,70 - 0,98** | **0,030** | **0,89** | **0,84 - 0,94** | **< 0,001** |
|  |  | Bilirubin (mg/L) |  |  |  |  |  |  |  |  |  |  |  |  |
|  |  | Tertile 1 | 1,00 | (ref) |  | 1,00 | (ref) |  | 1,00 | (ref) |  | 1,00 | (ref) |  |
|  |  | Tertile 2 | 1,65 | 0,53 - 5,18 | 0,389 | 1,07 | 0,70 - 1,64 | 0,761 | 2,21 | 0,82 - 6,00 | 0,119 | 1,05 | 0,76 - 1,46 | 0,762 |
|  |  | Tertile 3 | 3,49 | 0,98 - 12,45 | 0,054 | 1,14 | 0,76 - 1,71 | 0,526 | 3,29 | 0,95 - 11,31 | 0,059 | 1,18 | 0,86 - 1,60 | 0,299 |
|  |  | Haemoglobin (g/dL) | 0,94 | 0,79 - 1,11 | 0,469 | 0,95 | 0,89 - 1,01 | 0,117 | 0,90 | 0,77 - 1,05 | 0,187 | **0,89** | **0,85 - 0,93** | **< 0,001** |
|  |  | Arterial blood lactates (mmol/l) |  |  |  |  |  |  |  |  |  |  |  |  |
|  |  | Tertile 1 | 1,00 | (ref) |  | 1,00 | (ref) |  | 1,00 | (ref) |  | 1,00 | (ref) |  |
|  |  | Tertile 2 | 0,96 | 0,35 - 2,67 | 0,940 | 1,28 | 0,88 - 1,86 | 0,204 | 0,92 | 0,33 - 2,57 | 0,881 | 1,08 | 0,82 - 1,43 | 0,565 |
|  |  | Tertile 3 | 0,74 | 0,28 - 1,92 | 0,534 | **1,78** | **1,20 - 2,63** | **0,004** | 1,03 | 0,42 - 2,48 | 0,954 | **1,39** | **1,04 - 1,86** | **0,028** |
|  |  | ASAT (IU/L) |  |  |  |  |  |  |  |  |  |  |  |  |
|  |  | Tertile 1 | 1,00 | (ref) |  | 1,00 | (ref) |  | 1,00 | (ref) |  | 1,00 | (ref) |  |
|  |  | Tertile 2 | 0,65 | 0,15 - 2,72 | 0,554 | 1,21 | 0,79 - 1,85 | 0,393 | 0,91 | 0,23 - 3,52 | 0,891 | 1,23 | 0,89 - 1,70 | 0,213 |
|  |  | Tertile 3 | 1,19 | 0,33 - 4,33 | 0,794 | 1,47 | 0,97 - 2,23 | 0,071 | 1,43 | 0,40 - 5,10 | 0,578 | 1,28 | 0,92 - 1,78 | 0,140 |
|  |  | ALAT (IU/L) |  |  |  |  |  |  |  |  |  |  |  |  |
|  |  | Tertile 1 | 1,00 | (ref) |  | 1,00 | (ref) |  | 1,00 | (ref) |  | 1,00 | (ref) |  |
|  |  | Tertile 2 | 0,61 | 0,12 - 3,09 | 0,547 | 1,30 | 0,86 - 1,98 | 0,213 | 0,92 | 0,19 - 4,41 | 0,913 | 1,12 | 0,82 - 1,54 | 0,479 |
|  |  | Tertile 3 | 0,59 | 0,12 - 2,96 | 0,524 | 1,32 | 0,87 - 2,01 | 0,194 | 0,85 | 0,18 - 3,98 | 0,834 | 1,14 | 0,83 - 1,57 | 0,424 |
|  |  | BNP or NT-proBNP (pg/mL) |  |  |  |  |  |  |  |  |  |  |  |  |
|  |  | Tertile 1 | 1,00 | (ref) |  | 1,00 | (ref) |  | 1,00 | (ref) |  | 1,00 | (ref) |  |
|  |  | Tertile 2 | *not computed* | | | 1,40 | 0,82 - 2,36 | 0,215 | 2,29 | 0,47 - 11,06 | 0,303 | 1,39 | 0,95 - 2,04 | 0,088 |
|  |  | Tertile 3 | **3,22** | **1,03 - 10,07** | **0,044** | **1,85** | **1,12 - 3,07** | **0,017** | **3,57** | **1,14 - 11,23** | **0,029** | **1,87** | **1,29 - 2,71** | **0,001** |
|  |  | CRP (mg/L) |  |  |  |  |  |  |  |  |  |  |  |  |
|  |  | Tertile 1 | 1,00 | (ref) |  | 1,00 | (ref) |  | 1,00 | (ref) |  | 1,00 | (ref) |  |
|  |  | Tertile 2 | 1,04 | 0,19 - 5,87 | 0,962 | 1,97 | **1,15 - 3,37** | **0,013** | 0,80 | 0,15 - 4,25 | 0,796 | **1,61** | **1,09 - 2,38** | **0,017** |
|  |  | Tertile 3 | 2,23 | 0,48 - 10,35 | 0,308 | 2,04 | **1,20 - 3,47** | **0,008** | 2,23 | 0,57 - 8,70 | 0,247 | **2,10** | **1,44 - 3,06** | **< 0,001** |
|  | Baseline echography | |  |  |  |  |  |  |  |  |  |  |  |  |
|  |  | LVEF (for 10%) | **0,74** | **0,56 - 0,98** | **0,037** | **0,84** | **0,74 - 0,96** | **0,008** | **0,73** | **0,56 - 0,95** | **0,019** | **0,89** | **0,81 - 0,97** | **0,009** |
|  |  | Severe mitral regurgitation | 2,25 | 0,52 - 9,71 | 0,275 | 1,04 | 0,68 - 1,58 | 0,850 | 3,33 | 0,99 - 11,24 | 0,052 | 1,22 | 0,91 - 1,65 | 0,189 |
|  |  | Severe aortic stenosis | **5,23** | **1,17 - 23,35** | **0,030** | 1,18 | 0,65 - 2,14 | 0,589 | **5,41** | **1,21 - 24,19** | **0,027** | 1,28 | 0,82 - 1,99 | 0,274 |
|  |  | Severe aortic regurgitation | 1,17 | 0,16 - 8,79 | 0,879 | 1,75 | 0,56 - 5,49 | 0,336 | 1,03 | 0,14 - 7,69 | 0,978 | 0,87 | 0,28 - 2,71 | 0,807 |
|  | ACE : Angiotensin-Converting Enzyme. ALAT : ALanine AminoTransferase. ARB : Angiotensin-Receptor Blocker. ASAT : ASpartate AminoTransferase. BMI : Body Mass Index. CABG : Coronary Artery Bypass Graft. CICU : Cardiologic Intensive Care Unit. COPD : Chronic Obstructive Pulmonary Disease. CRP : C-Reactive Protein. DBP : Diastolic Blood Pressure. IQR : InterQuartile Range. LVEF : Left Ventricular Ejection Fraction. PCI : Percutaneous Coronary Intervention. SD : Standard Deviation. SBP : Systolic Blood Pressure. SD : Standard Deviation. | | | | | | | | | | | | | |
